# Direct Interaction between Selenoprotein P and Tubulin

**DOI:** 10.3390/ijms150610199

**Published:** 2014-06-06

**Authors:** Xiubo Du, Shi Qiu, Zhi Wang, Ruoran Wang, Chao Wang, Jing Tian, Qiong Liu

**Affiliations:** 1 Department of Marine Biology, Shenzhen Key Laboratory of Marine Biotechnology and Ecology, Shenzhen University, Shenzhen 518060, China; E-Mails: xiubo.du@gmail.com (X.D.); wangzhi.szu@gmail.com (Z.W.); jing.tianjingtj@gmail.com (J.T.); 2 College of Life Sciences, Shenzhen Key Laboratory of Microbial Genetic Engineering, Shenzhen University, Shenzhen 518060, China; E-Mails: qiuqiushi@hotmail.com (S.Q.); wangruoran@hotmail.com (R.W.); raulw2003@163.com (C.W.)

**Keywords:** selenoprotein P (SelP), tubulin, protein-protein interaction, yeast two-hybrid system, fluorescence resonance energy transfer (fret)

## Abstract

Selenium (Se), an essential trace element for human health, mainly exerts its biological function via selenoproteins. Among the 25 selenoproteins identified in human, selenoprotein P (SelP) is the only one that contains multiple selenocysteines (Sec) in the sequence, and has been suggested to function as a Se transporter. Upon feeding a selenium-deficient diet, mice lacking SelP develop severe neurological dysfunction and exhibit widespread brainstem neurodegeneration, indicating an important role of SelP in normal brain function. To further elucidate the function of SelP in the brain, SelP was screened by the yeast two-hybrid system from a human fetal brain cDNA library for interactive proteins. Our results demonstrated that SelP interacts with tubulin, alpha 1a (TUBA1A). The interaction between SelP and tubulin was verified by fluorescence resonance energy transfer (FRET) and co-immunoprecipitation (co-IP) assays. We further found that SelP interacts with the *C*-terminus of tubulin by its His-rich domain, as demonstrated by FRET and Isothermal Titration Calorimetry (ITC) assays. The implications of the interaction between SelP and tubulin in the brain and in Alzheimer’s disease are discussed.

## 1. Introduction

Selenium (Se) is an essential trace element for human health, the deficiency of which is closely related to the incidence of some diseases, including neurodegenerative diseases [1,2,3]. Se exerts its biological function mainly through selenoproteins in which Se is present in the form of selenocysteine (Sec) that is encoded by a traditional stop codon (UGA) in the open reading frame. Sec incorporation in protein at UGA codons requires cis-acting mRNA secondary structures and several specialized trans-acting factors [4], which makes it very difficult to produce selenoproteins in heterologous expression systems and thus has limited the structural and functional study of selenoproteins. The SelP gene in different mammalian species contains 10–12 in frame UGA codons and two SECIS (selenocysteine insertion sequence) elements in the 3' untranslated region (3'-UTR), which distinguishes SelP from all other selenoproteins. Four isoforms have been found in human SelP that contain 1, 2, 6, and 10 Sec residues, respectively [5]. SelP is expressed in most tissues, but with highest levels produced in liver, and secreted either into plasma or interstitial fluids. Apolipoprotein E receptor-2 (apoER2), a member of the lipoprotein receptor family, binds SelP and mediates its uptake into testis and retention of its selenium by the brain [6]. Megalin, another lipoprotein receptor, facilitates uptake of filtered SelP into proximal tubule cells of the kidney [7].

Deletion of *SelP* results in increased excretion of selenium in the urine and decreased levels of selenium in plasma, brain, testis and kidney, as well as in the whole body [8,9]. Thus, SelP serves in homeostasis and distribution of selenium. Based on the distribution of selenium, SelP is divided into two domains. A larger *N*-terminal domain contains 1 Sec and 7 Cys and a smaller *C*-terminal domain consists of 9 Sec and 10 Cys. Thus, the *C*-terminal domain contains 90% of its selenium, raising the possibility that this domain plays a role in selenium transport. The *C*-terminus mouse SelP was found to be necessary for the supply of selenium to the brain and testis, but not for the maintenance of the whole-body selenium [10]. The *N*-terminal domain has two potential redox motifs: ^40^UXXC^43^ and ^153^CXXC^156^, and exhibits enzyme activity. Recently, Kurokawa *et al.* purified 11 SelP *N*-terminal fragments from the urine of *megalin*^−/−^ mice, and found that these fragments all terminated at 11 sites between residues 183 and 208. They are TrxR1 substrates, catalyzing NADPH oxidation when coupled with H_2_O_2_ or *tert-*butylhydroperoxide as the terminal electron acceptor, as were also the full length SelP and its *C*-terminal truncated form SelP^Δ^^240–361^ [11]. These results confirm that SelP is a multi-functional protein and suggest that the first selenocysteine residue is the active site of the enzyme and the remaining nine residues function as a selenium source.

Expression of SelP is upregulated in an age-dependent manner [12]. Moreover, SelP was found to be co-localized with both senile plaques and neurofibrillary tangles in the postmortem tissue from individuals with the hallmark lesions of Alzheimer’s disease (AD) [13]. The direct association of SelP expression with the pathology of AD suggests that this protein is involved in the response or progression of the disorder. Knockdown of SelP rendered Neuro-2a (N2A) cells more sensitive to the toxicity of amyloid-beta peptide (Aβ) [14]. However, the exact function and mechanism of SelP in AD prevention remain unknown. In order to investigate the biological function of SelP in the brain, the interactive protein of SelP was screened by the yeast two-hybrid system and this interaction was verified by the fluorescence resonance energy transfer (FRET) technique, followed by coimmunoprecipitation (Co-IP) assays. Tubulin, alpha 1a (TUBA1A) was finally found to interact with SelP in human brain, the implications of which in AD are discussed.

## 2. Results and Discussion

### 2.1. Screening an Interacting Protein of SelP from the Human Fetal-Brain cDNA Library

**Figure 1:**
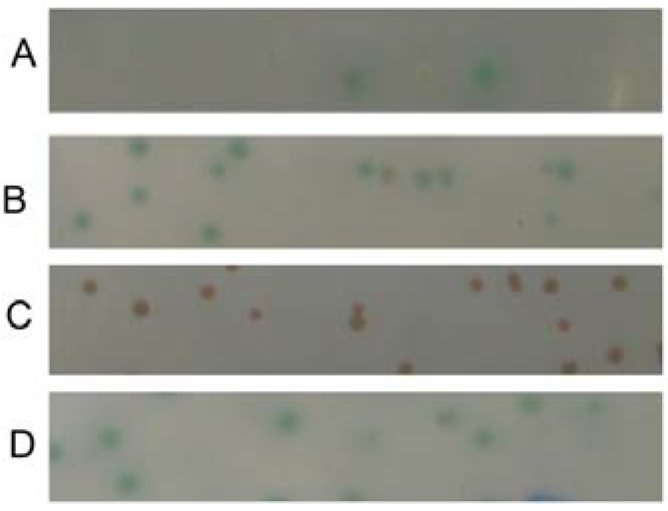
Yeast two-hybrid screening of the human fetal brain cDNA library using the Sec to Cys mutant of SelP (SelP') gene as a bait. (**A**) Plasmids carrying the fetal brain cDNA library were co-transformed into the NpGBKT7-*SelP'*-containing yeast and screened by the selection plate for blue colonies; Y2H Gold yeast cells in (**B**)–(**D**) were co-transformed with plasmids NpGBKT7-*SelP'* and pACT2-*Tub* to verify the interaction between SelP' and tubulin (**B**); or with plasmids pGBKT7-Lam and pADT7-T as the negative control (**C**); or with plasmids pGBKT7-p53 and pADT7-T as the positive control (**D**), followed by selection on SD/-Trp/-Leu/-His/-Ade/X-α-gal/Aba plates.

Due to the absence of selenoprotein synthesis machinery in yeast cells, the ten Sec-coding TGA codons in *selp* were changed to Cys-coding TGC using sited directed mutagenesis to generate the *selp'* gene, which was then inserted into the vector NpGBKT7 to get the bait plasmid NpGBKT7-*selp'* (BD (binding domain) plasmid). Before screening, NpGBKT7-*selp'* and the empty plasmid pGADT7 were co-transformed into AH109 yeast cells and no self-activation was observed (data not shown). The NpGBKT7-*selp'*-containing yeast was then used to screen the fetal brain cDNA library. On the corresponding selection plates (SD/-Ade/-His/-Leu/-Trp) containing X-α-Gal and aureobasidin A (Aba), several colonies were grown (Figure 1A), the plasmids of which were extracted and transformed into the *Escherichia coli* (*E. coli*) Top 10 cells separately, to screen for colonies carrying the gene of the interactive protein (AD (active domain) plasmid). The BD plasmid and the screened AD plasmid were then re-transformed into Y2H Gold yeast cells, followed by the plate selection (SD/-Trp/-Leu/-His/-Ade/X-α-gal/Aba). The AD plasmids in two positive colonies following re-transformation were subjected to sequencing and bioinformatics analysis with National Center for Biotechnology Information (NCBI)’s non-redundant (nr) protein database. One of them was identified to be *Homo sapiens* tubulin, alpha 1a (TUBA1A).

### 2.2. Verification of the Interaction between SelP' and Tubulin by Fluorescence Resonance Energy Transfer (FRET)

**Figure 2:**
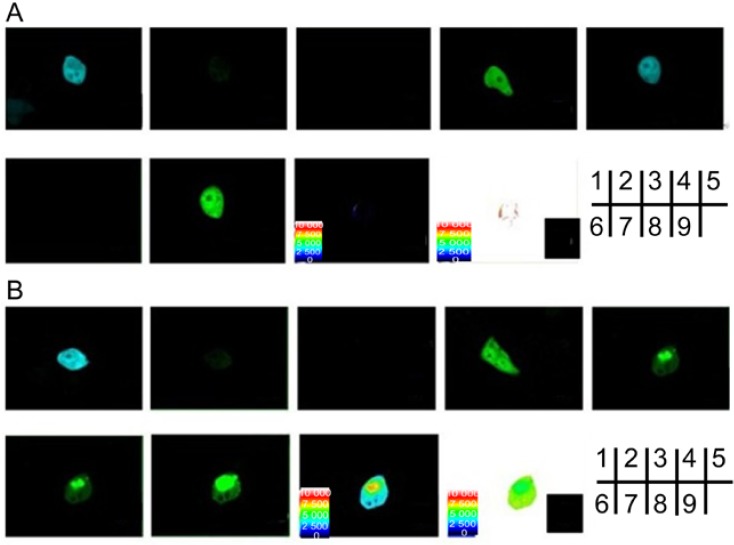
Verification of SelP' and α-tubulin interaction by the sensitized emission method of fluorescence resonance energy transfer (FRET). HEK293T cells were transfected respectively or together with (**A**) the vacant plasmids pECFP-C1 and pEYFP-C1 as negative controls; (**B**) pECFP-C1-*SelP'* and pEYFP-C1-*Tub*. (1) and (2) Donor cells were transfected with CFP plasmids, excited at 405 nm, and imaged at CFP channel (1) or YFP channel (2); (3) and (4) Receptor cells were transfected with YFP plasmids and imaged at YFP channel, where the cells were excited at 405 nm (3) or 515 nm (4); (5)–(9) Cells were co-transfected with CFP and YFP plasmids; (5) Cells were excited at 405 nm and imaged at CFP channel; (6) Cells were excited at 405 nm and imaged at YFP channel; (7) Cells were excited at 515 nm and imaged at YFP channel; (8) Diagram of the distance between donor and receptor. (9) FRET efficiency diagram.

To verify the interaction between SelP' and tubulin, FRET methods of sensitized emission and receptor photobleaching were performed. The coding sequences of SelP' and tubulin genes were inserted into the expression vectors containing the enhanced cyan fluorescence protein (CFP) and yellow fluorescence protein (YFP), respectively. HEK293T cells were cotransfected by the plasmids pECFP-C1-*selp'* and pEYFP-C1-*tub*. For the sensitized emission assay, the energy transfer efficiency between CFP-SelP' (donor) and YFP-Tub (receptor) was calculated to be 21.3% ± 7.2% (*n* = 5), and the distance between the donor and receptor was calculated to be 6.8 ± 0.4 nm (*n* = 5) (Figure 2B, Table 1). Cells co-transfected with empty vectors pECFP-C1 and pEYFP-C1 (Figure 2A) were used as control, which showed an average FRET efficiency of 1.4%, indicating no interaction between ECFP and EYFP.

**Table 1:** Fluorescence resonance energy transfer (FRET) efficiency and distance of the region of interest (ROI) from the cells co-transfected with pECFP-C1-*SelP'* and pEYFP-C1-*Tub* determined by sensitized emission method.

Cell Number	FRET’s Efficiency *E* (%)	Distance between Donor and Acceptor *r* (nm)
1	19.7	6.981
2	18.7	6.884
3	32.6	6.095
4	17.7	7.222
5	18.0	6.849
Average	21.34	6.806

**Figure 3:**
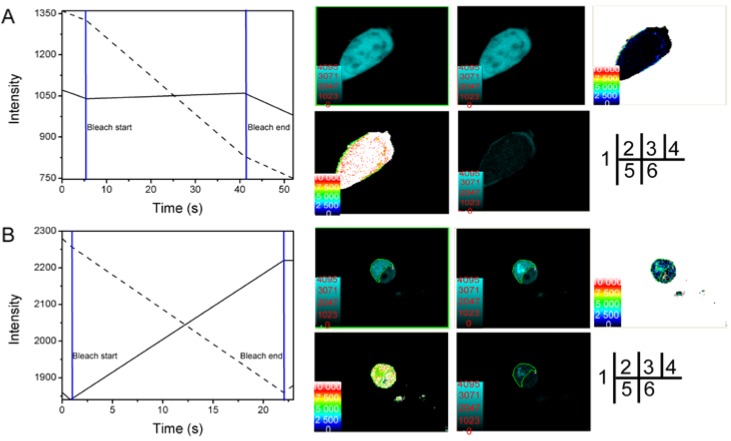
Verification of SelP' and α-tubulin interaction by the receptor photobleaching method of FRET. HEK293T cells were co-transfected with the empty plasmids pECFP-C1 and pEYFP-C1 as a negative control (**A**) or co-transfected with pECFP-C1-*SelP'* and pEYFP-C1-*Tub* for sample tests (**B**). (1) Photobleaching curves (solid lines for donor fluorescence and dashed lines for receptor fluorescence). The region of interest (ROI) was bleached at 515 nm; (2) The fluorescence images of donors before bleaching; (3) The fluorescence images of donors after bleaching; (4) Donor fluorescence increments before and after bleaching; (5) Diagram of the distance between donor and receptor; (6) FRET efficiency diagram.

**Figure 4:**
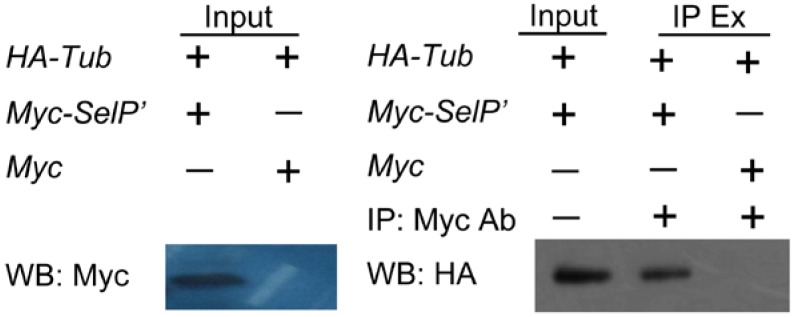
Verification of SelP' and α-tubulin interaction by co-immunoprecipitation assay. HEK293T cells were co-transfected with plasmids pCMV5.0-HA-*Tub* and pCDNA3.1-Myc-*SelP'* (or pCDNA3.1-Myc as the negative control). The supernatants of cell lysates were immunoprecipitated (IP) with anti-Myc antibody and analyzed by Western blot (WB) using anti-HA antibody. The supernatant of cell lysate, named Input, was used as a positive control for WB analysis probed with both anti-Myc and anti-HA antibodies. IP Ex: IP extract.

**Figure 5:**
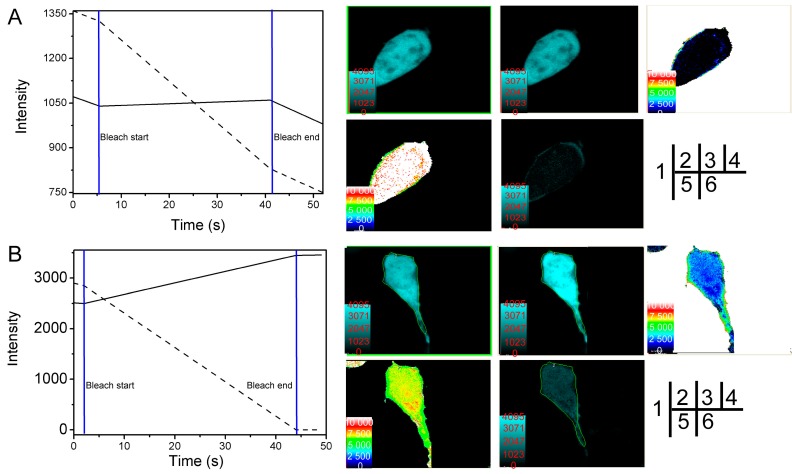
Verification of SelP-H and Tub-C interaction by the receptor photobleaching method of FRET. HEK293T cells were co-transfected with the empty plasmids pECFP-C1 and pEYFP-C1 as a negative control (**A**) or co-transfected with pECFP-C1-*selp-h* and pEYFP-C1-*tub-c* for sample tests (**B**). (1) Photobleaching curves (solid lines for donor fluorescence and dashed lines for receptor fluorescence). The region of interest (ROI) was bleached at 515 nm; (2) The fluorescence images of donors before bleaching; (3) The fluorescence images of donors after bleaching; (4) Donor fluorescence increments before and after bleaching; (5) Diagram of the distance between donor and receptor; (6) FRET efficiency diagram.

**Figure 6:**
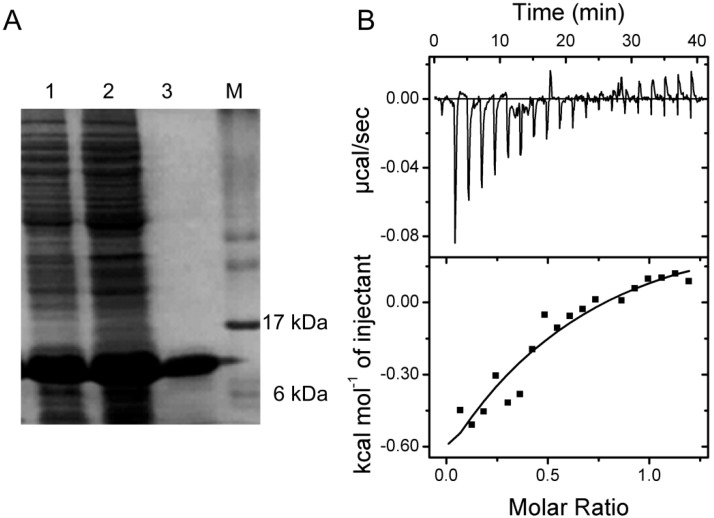
(**A**) SDS-PAGE image showing the purification of Tub-C by Ni^2+^ affinity chromatography. Lane 1, cell lysate; Lane 2, elutes with 60 mM imidazole from the column; Lane 3, elutes with 0.5 mM imidazole from the column, corresponding to purified Tub-C; (**B**) Isothermal titration calorimetric analyses of the binding of Tub-C to SelP-H. **Top**: raw data; **Bottom**: plot of integrated heat *versus* Tub-C/SelP-H molar ratio. The solid curve represents the best fit with sequential binding model.

Results from the acceptor photobleaching experiments showed that fluorescence of the CFP-SelP' donor was significantly increased after the receptor was bleached (Figure 3B-4), which was not observed in control cells (Figure 3A-4). The distance between CFP-SelP' donor and YFP-Tub receptor was estimated to be 7.5 ± 0.5 nm (*n* = 11) (Figure 3B-6, Table 2). The energy transfer efficiency between the two proteins was calculated to be 21.8% ± 5.6% (*n* = 11) (Figure 3B-5, Table 2). The FRET efficiency of control cells co-transfected with empty vectors pECFP-C1 and pEYFP-C1 was calculated to be 3.4% (*n* = 3) (Figure 3A-6), and the distance was estimated to be 9.08 nm (*n* = 3, Figure 3A-5). Results from FRET assays confirmed the interaction between SelP' and tubulin.

**Table 2:** FRET efficiency and distance of the ROI from the cells co-transfected with pECFP-C1-*SelP'* and pEYFP-C1-*Tub* determined by receptor photobleaching method.

Cell Number	FRET Efficiency *E* (%)	Distance between Donor and Acceptor *r* (nm)
1	17.6	7.794
2	14.1	8.239
3	12.2	8.816
4	31.5	6.634
5	20.0	7.595
6	26.7	7.293
7	19.2	7.845
8	17.9	7.744
9	35.6	6.341
10	20.2	7.551
11	25.0	6.856
Average	21.81	7.519

### 2.3. Verification of the Interaction between SelP' and Tubulin by Co-Immunoprecipitation Assay

To further confirm the interaction between SelP' and tubulin, co-immunoprecipitation (Co-IP)assays were performed. The constructed plasmids pCDNA3.1-Myc-*selp'* and pCMV5.0-HA-*tub* were co-transfected into HEK 293T cells. An antibody against Myc was used to immunoprecipitate (IP) Myc-tagged SelP' from cell extracts. The isolated proteins were analyzed by western blot using an anti-HA antibody. A specific association between Myc-tagged SelP' and HA-tagged tubulin is shown in lane 2 of Figure 4, further confirming the interaction between SelP' and tubulin in mammalian cells.

### 2.4. Studying the Interactive Domains between SelP' and Tubulin by FRET

α-tubulin, together with β-tubulin and several microtubule associated proteins, polymerize to form microtubules, which are involved in a number of critical cellular processes, such as the determination of cell shape, chromosome segregation, intracellular transport of vesicles and organelles, and cell migration. α-tubulin is organized into three domains, namely the *N*-terminal domain (amino acid residues 1–205) involved in nucleotide binding, the intermediate domain (amino acid residues 206–384), and the *C*-terminal domain (amino acid residues 385 to the *C*-terminus). The *C*-terminus of α-tubulin is involved in multiple aspects of the regulation of microtubule assembly. A recent study revealed that this region interacts with several cationic molecules, including Tau, polyamines and calcium [15], mainly by its highly negatively charged tail of about 20 amino acids. The tail protrudes from the surface of microtubules, binds cationic molecules by charge-charge interaction. In SelP, there are three domains (*N*-terminal region, *C*-terminal region and His-rich domain, SelP-H) predicated on the surface of the protein by PROFace program. SelP encodes two His-rich regions, located at residue 204–217 and residue 244–250, respectively. The functions of the *N*-terminus and *C*-terminus were previously reported to encompass oxidant defense and Se homeostasis [10,11]. However, the His-rich domain was less studied. Recently, we found SelP-H bound transition metal ions tightly and importantly regulated metal-mediated Aβ_42_ aggregation *in vitro*, ROS generation and toxicity in living cells [16,17]. Here, we propose the basic His-rich motifs of SelP-H may interact with the acidic tail of α-tubulin via charge-charge interaction.

The interaction between SelP-H and the *C*-terminal domain of α-tubulin (the last 42 residues, named Tub-C) was first tested by FRET with the method of acceptor photobleaching. Plasmids pECFP-C1-*selp-h* and pEYFP-C1-*tub-c* were constructed and cotransfected into HEK293T cells for FRET assay. Fluorescence of the CFP-*SelP-H* donor was significantly increased after the receptor was bleached (Figure 5), which was not observed in the control group. The energy transfer efficiency and distance between CFP-SelP-H donor and YFP-*Tub-C* receptor were estimated to be 22.8% ± 5.4% and 7.4 ± 0.5 nm (*n* = 12), respectively. The FRET experiments preliminarily confirmed the interaction between SelP-H and Tub-C.

### 2.5. Verification of the Interaction between SelP-H and Tub-C by Isothermal Titration Calorimetry (ITC)

To further confirm and thermodynamically characterize the interaction between SelP-H and Tub-C, Tub-C (1.3 mM) were titrated into 0.228 mM SelP-H and the calorimetric changes were monitored using iTC200 (GE Healthcare, Northampton, MA, USA). SelP-H was expressed and purified as described previously [17]. To express Tub-C, a DNA fragment encoding the last 42 residues of α-tubulin was amplified and inserted into pET15b vector to generate pET15b-*tub-c* plasmid. Tub-C protein was expressed from BL21 transformed with pET15b-Tub-C and purified with the aid of Ni^2+^-IMAC chromatography. The purified Tub-C migrated to around 10 kDa on a 12% SDS-polyacrylamide gel (Figure 6A).

Titration of Tub-C into SelP-H resulted in exothermic peaks, which eventually diminished to just the heat of dilution after 10 injections (Figure 6B). The results were analyzed with the models using the Origin Software package (Microcal, Northhampton, MA, USA). A nonlinear least squares method was used to obtain the best fit parameters for the number of binding sites *n*, the association constant *K*, and the change of enthalpy Δ*H* and entropy Δ*S*. Several binding models including one set of sites model (*n* identical sites, 3 parameters), two sets of sites model (independent sites, 6 parameters) and sequential binding model, were used in the fitting. The goodness-of-fit with different models were compared according to the calculated χ^2^ value, and sequential binding model (with *n* = 2) was found to give the best fit. Analysis of the binding isotherm using sequential binding model (with *n* = 2) approximated the association constants to be *K*_1_ = 2.1 × 10^3^ ± 1.5 × 10^3^ M^−1^ and *K*_2_ = 1.5 × 10^3^ ± 1.2 × 10^3^ M^−1^. The Δ*H* and Δ*S* were estimated to be Δ*H*_1_ = −1.8 ± 0.9 kcal·mol^−1^, Δ*H*_2_ = 6.9 ± 2.4 kcal·mol^−1^ and Δ*S*_1_ = 9.1 cal·mol^−1^·K, Δ*S*_2_ = 37.8 cal·mol^−1^·K, respectively.

Co-localization of two proteins is a prerequisite for their interaction. Tubulin is an intracellular protein. SelP is mostly expressed in the liver as a glycosylated protein and then secreted into the plasma, but is also produced by other tissues, albeit in lesser quantities [18]. In the brain, the primary source of SelP is astrocytes, while SelP was also detected inside the neurons by immunohistochemistry [19]. Steinbrenner *et al.* demonstrated primary human astrocytes and the human astrocytoma cell line MOG-G-CCM express SelP as an unglycosylated protein, which is not secreted and protects astrocytes against *tert*-butyl hydroperoxide (*t*-BHP)-induced oxidative damage [20]. Furthermore, plasma SelP is proven to be taken up by Sertoli cell [6] in the testis and neurons in the brain [21], and by proximal convoluted tubule (PCT) cells in the kidney [7], with apoliporptein E receptor-2 (apoER2) and megalin-mediated endocytosis of the protein, respectively. SelP-containing vesicles were identified in Sertoli cells and PCT cells by immunocytochemistry [6,7]. Therefore, although primarily extracellular, SelP might possibly be intracellular under some circumstances and provide it opportunities to interact with tubulin. The association of SelP and tubulin implied that SelP may play certain roles in regulating the dynamic property of microtubules, which is important for many microtubule-mediated cellular events such as cell division and migration; or maintaining the stability of microtubules, which is important for intracellular transport and the maintenance of cell polarity.

Microtubules (MTs) are the key components of the cytoskeleton of eukaryotic cells and have an important role in various cellular functions such as intracellular migration and transport, cell shape maintenance, polarity, cell signaling and mitosis [22]. Microtubules are composed of two globular protein subunits, α- and β-tubulin. These two subunits combine to form an α, β-heterodimer which then assembles in a filamentous tube-shaped structure. The importance of MT dynamics and stability is underscored by the critical role Tau protein plays in MT-associated stabilization *versus* the dysfunction seen in Alzheimer’s disease, frontotemporal dementia and other tauopathies. In AD, MT associated protein Tau was hyperphosphorylated, resulting in the collapse of cytoskeleton and the formation of neurofibrillary tangles (NFT). MTs are disrupted in the brains of patients and animal models of tauopathies. For example, MT density was reduced in the hippocampal neurons of transgenic mice expressing the V337M mutant of human Tau and in the spinal ventral root axons of transgenic mice expressing the smallest isoform of human Tau [23,24]. Furthermore, administration of the MT-stabilizing agents such as paclitaxel and epothilone D to human Tau-expressing mice resulted in improved MT density and axonal integrity [25], as well as enhanced cognitive performance [26]. Previously, van Eersel reported sodium selenate reduced Tau phosphorylation, completely abrogated NFT formation in Tau transgenic mouse, improved its contextual memory and motor performance, and finally prevented its neurodegeneration [27]. Consistently, our lab found selenomethionine ameliorated cognitive decline, reduced Tau hyperphosphorylation, and reversed synaptic deficit in the triple transgenic mouse model of AD [28]. The interaction between SelP and tubulin suggested that SelP may supply selenium to inhibit the abnormal phosphorylation of Tau and the formation of neurofibrillary tangles, considering the Se transporting function of SelP.

Growing evidence has also shown that oxidative stress may have a role in the hyperphosphoryaltion and polymerization of Tau. Increase of ROS results in the dyshomeostasis of calcium, promotes calcium influx, which actives calcium-dependent protein kinases (such as PKC) and causes the imbalance between protein phosphatases and protein kinases, thus finally increases the abnormal phosphorylation of Tau [29]. In addition, oxidation of fatty acids, which is found to be elevated in AD brains, was reported to facilitate the polymerization of Tau [30]. The high content of Sec makes SelP to have antioxidant properties *in vivo*, especially considering the presence of the potential redox motifs (^40^UXXC^43^ and ^153^CXXC^156^) in the *N*-terminal domain of SelP. SelP was reported to inhibit both radiation induced and 1-(4-Chlorophenyl)-benzo-2,5-quinone (4-ClBQ) induced ROS production [31,32]. The interaction between SelP and tubulin therefore might attenuate ROS-mediated Tau phosphorylation and NFT formation. In addition, SelP may also function as a stabilizer of microtubule. It would be of interest for future work to investigate the exact mechanism of the function of SelP in AD.

## 3. Experimental Section

### 3.1. Materials

Matchmaker™ Gold yeast two-hybrid system, yeast strains Y2HGold and AH109, plasmids pACT2 and NpGBKT7, human fetal brain cDNA library (using pACT2 as the vector), anti-fungal antibiotic aureobasidin A (Aba), and synthetic dropout (SD) medium lack some certain nutrients (that is, SD/-Leu, SD/-Trp, SD/-Ade-Trp-Leu, and SD/-Ade-His_3_-Trp-Leu) were purchased from Clontech (Mountain View, CA, USA). Human embryonic kidney 293T (HEK293T) cells and *E. coli* strain Top 10 were kept in our lab. Myc and HA monoclonal antibodies were from SANTA CRUZ (Dallas, TX, USA). Horseradish peroxidase (HRP)-goat anti-mouse IgG was purchased from Invitrogen (Grand Island, NY, USA). Fluorescence protein-containing vectors pECFP-C1 and pEYFP-C1 were kindly provided by Professor Shengli Tian from Shenzhen University. All primers were synthesized by Invitrogen (Grand Island, NY, USA).

### 3.2. Gene Amplification and Plasmid Construction

The truncated SelP gene without signal peptide (CDS position: 58–1140) was PCR-amplified from the human hepatocytes L-02 (GIBCO, Grand Island, NY, USA). Ten Sec-encoding TGAs are located inside the open reading frame (ORF) at the following positions: 175–177; 898–900; 952–954; 988–990; 1033–1035; 1054–1056; 1099–1101; 1105–1107; 1126–1128; 1132–1134. Those in-frame TGAs were multi-site-directly mutated to Cys-encoding TGCs using the method developed by our lab [33], with the primers listed in Table 3. The obtained mutant *selp* gene was named as *selp'* and inserted into the following plasmids: NpGBKT7, pECFP-C1 and pCDNA3.1-Myc, to generate NpGBKT7-*SelP'*, pECFP-C1-S*elP'* and pCDNA3.1-Myc-*SelP'* for Yeast two-hybrid, FRET and Co-IP experiments, respectively.

**Table 3:** Primers used for SelP mutation.

Number	Sequence
F1	5'-ATGGCCATTACGGCCGAGAGCCAGGACCAAAGCTCCTTATGTAAGCAACC-3'
R1	5'-ATGGCCGAGGCGGCCTTAGTTTGAAGGGCATTCGCACTTTTTTGCCTGATTC-3'
F2	5'-TCTTCAAGCCAGC**TGC**TACCTGTGCATACTGC-3'
R2	5'-**GCA**GCTGGCTTGAAGAAGAGCAACCACAGTCAC-3'
F3	5'-TGGCTCCTAGGAGC**TGC**TGCTGCCATTGTCGAC-3'
R3	5'-**GCA**GCTCCTAGGAGCCAACTCTGAATCTGTGG-3'
F4	5'-TCTGCAATCACC**TGC**CAGTGTAAAGAAAACC-3'
R4	5'-**GCA**GGTGATTGCAGACCCTGTTTTTTCAAAT-3'
F5	5'-ATCTTTATGTAGC**TGC**CAGGGACTTCGGGCAG-3'
R5	5'-**GCA**GCTACATAAAGATGGGAGGTTTTCTTTAC-3'
F6	5'-**TGC***CGTTTGCCTCCAGCAGCC***TGC**CAAATAAGTCAGCAGCTTAT-3'
R6	5'-**GCA**GGCTGCTGGAG**GCA**AACGGCACTGACAAGATTCAGTTATG-3'
F7	5'-AGTGCCAGT **TGC**CGC**TGC**AAGAATCAGGC-3'
R7	5'-**GCA**GCG**GCA**ACTGGCACTGGCTTCTGTGG-3'

The in-frame Sec-encoding TGAs were mutated to Cys-encoding TGCs and highlighted in bold.

### 3.3. Library Screening via the Yeast Two-Hybrid System

Yeast transformation and two-hybrid screening were performed using the bait plasmid NpGBKT7-SelP' to screen the human fetal brain cDNA library, following the procedures described in the Yeastmaker™ Yeast Transformation System 2 User Manual and the Matchmaker™ GAL4 Two-Hybrid System 3 & Libraries User Manual (Clontech, Mountain View, CA, USA). The screened prey plasmid was cotransformed with the bait plasmid into yeast for re-transformation verification.

### 3.4. Mammalian Cell Culture and Plasmid Transfection

HEK293T cells were cultured in Dulbecco’s Modified Eagle’s Medium (DMEM) supplemented with 10% fetal bovine serum and maintained at 37 °C in 5% CO2. Before transfection, the full culture medium was changed to serum-free medium. Plasmids and the DNA-MATE transfection reagent were both diluted with phosphate buffer saline (PBS) and then mixed thoroughly. The mixture was left at room temperature for 30 min, then added into the cell culture with serum-free DMEM, and incubated for 4 h at 37 °C in 5% CO_2_. Cells were then cultured in full DMEM medium for another 24–48 h for the following FRET or co-IP assays. To perform FRET experiments, 2–4 × 10^5^ cells were seeded in 35 mm culture dishes and transfected respectively with 4 μg plasmids of pECFP-C1-*SelP'* and pEYFP-C1-*Tub*, or cotransfected with plasmids pECFP-C1-*SelP'* and pEYFP-C1-*Tub*, using pECFP-C1 and pEYFP-C1 as control groups.

### 3.5. Fluorescence Resonance Energy Transfer (FRET) Analyses

To perform FRET experiments, 2–4 × 10^5^ HEK293T cells were seeded in 35 mm culture dishes and transfected respectively with 4 μg plasmids of pECFP-C1-*SelP'* (or pECFP-C1-*selp-h*) and pEYFP-C1-*Tub* (or pEYFP-C1-*tub-c*), or co-transfected with plasmids pECFP-C1-*SelP'* (or pECFP-C1-*selp-h*) and pEYFP-C1-*Tub* (or pEYFP-C1-*Tub-c*), using pECFP-C1 and pEYFP-C1 as control groups. The fluorescent images were acquired through the laser confocal microscope 48 h after cell transfection.

#### 3.5.1. Acceptor Bleaching Method

The acceptor signal was bleached in defined regions of interest (ROI) with 515 nm light at 95% laser power for 20–30 s. The change in donor (ECFP) fluorescence induced by acceptor photobleaching was quantified by comparing prebleach and postbleach images obtained by excitation at 405 nm. The acquired data was analysed using Olympus Fluoview FV1000 Toolbox software (Olympus, Tokyo, Japan). FRET efficiency was calculated as 1-I_prebleaching_/I_postbleaching_, where I_prebleaching_ and I_postbleaching_ are the intensities of ECFP before and after the bleach in defined ROIs. Distance (*r*) was calculated as R_0_(1/E-1)^1/6^, where R_0_ value for the pairing of CFP/YFP is 5.2767 nm. The background was determined by outlining a ROI in a region containing no cells. This background value was subtracted from each value obtained in the cells. The mean FRET intensities obtained in at least 20 ROIs from at least three different transfections were measured for each protein pair. As controls, FRET in cells transfected with the tags alone, that is pECFP-C1 and pEYFP-C1, was also studied.

#### 3.5.2. Sensitized Emission Method

Seven fluorescence images were collected as follows: cells transfected alone with pECFP-C1-SelP' and imaged at both CFP and YFP channels under 405-nm-excitation; cells transfected alone with pEYFP-C1-*Tub* and imaged at YFP channel under the excitation of 405 and 515 nm; cells co-transfected with pECFP-C1-*SelP'* and pEYFP-C1-*Tub*, and imaged at CFP channel under 405-nm-excitation or imaged at YFP channel under the excitation of 405 and 515 nm. The energy transfer efficiency and molecular distance between the donor and receptor were calculated by the software FV10-ASW2.1 Viewer (Olympus, Tokyo, Japan).

### 3.6. Verification by Co-Immunoprecipitation Assay

To perform co-IP assays, about 1 × 10^6^ HEK293T cells were seeded in 100 mm culture dishes and cotransfected with 24 μg plasmids of pCDNA3.1-Myc-*SelP'* and pCMV5.0-HA-*Tub*, using pCDNA3.1-Myc and pCMV5.0-HA-*Tub* as the control group. Forty-eight h after transfection, the cells were lysed in RIPA solution (Bonataike, Shenzhen, China). The lysates were centrifuged at 13,000 rpm for 30 min at 4 °C. The supernatants were collected and total protein amount was determined by the bicinchoninic acid (BCA) method. A portion of the supernatant corresponding to 1 mg of total proteins was mixed with protein G plus-agarose beads at 4 °C for 1 h to preclear the sample for minimizing nonspecific binding of proteins. Then the precleared supernatant was incubated with 2 μg of anti-Myc antibody for 1 h at 4 °C, followed by incubation with protein G-agarose beads overnight at 4 °C. The agarose beads were spinned down at 2000 rpm for 2 min, washed three times with RIPA lysis buffer and twice with PBS. The collected proteins were suspended in 2 × SDS-PAGE loading buffer, separated by SDS-PAGE and analyzed by Western blot using the primary antibody of anti-HA.

### 3.7. Isothermal Titration Calorimetry (ITC)

ITC measurements were performed on a MicroCal iTC-200 microcalorimeter (GE healthcare, Northampton, MA, USA) at 25 °C. The purified proteins (SelP-H and Tub-C) were subjected to three rounds of dialysis against Tris buffer (50 mM, pH 7.4). The proteins concentrations were determined by using BCA Protein Assay Kit with bovine serum albumin (BSA) as a standard. In a typical titration, 2 μL of 1.3 mM Tub-C was titrated into 200 μL of 228 μM SelP-H over 4 s with a 3-min interval between each injection. Twenty injections were made in total. The reaction solution was stirred at 1000 rpm. The heat of dilution, mechanical effects and non-specific interactions were accounted for by averaging the last three points of titration and the value was subtracted from all data points. Experiments were repeated three times under the same conditions.

## 4. Conclusions

The interactive protein of SelP in the human brain was investigated in this paper. A human fetal brain cDNA library was screened with SelP' using the yeast two-hybrid system. A new interactive protein of SelP' was identified and sequence analysis determined that it was α-tubulin. The interaction between SelP and tubulin was further verified by FRET with the methods of sensitized emission and receptor photobleaching, as well as co-IP assay. Next, we found the *C*-terminus of tubulin bound directly to the His-rich domain of SelP through FRET and ITC studies. In AD, the microtubule associated protein Tau is hyperphosphorylated and dissociated from microtubules, resulted in the collapse of the neuronal cytoskeleton, which could be significantly restored by the administration of selenate. The interaction between SelP and tubulin suggested that SelP may provide selenium to inhibit its abnormal phosphorylation or surpress ROS generation and thus attenuate ROS-mediated Tau phosphorylation and NFT formation. It would be of interest for future work to investigate the exact mechanism of the function of SelP in AD.

## References

[1] Rayman M.P. (2000). The importance of selenium to human health. Lancet.

[2] Brown K.M., Arthur J.R. (2001). Selenium, selenoproteins and human health: A review. Pub. Health Nutr..

[3] Liu Q., Tian J., Chen P., Yang S.L., Song Y. (2012). Selenium deficiency and Alzheimer’s disease. Chin. Bull. Life Sci..

[4] Forchhammer K., Leinfelder W., Bock A. (1989). Identification of a novel translation factor necessary for the incorporation of selenocysteine into protein. Nature.

[5] Burk R.F., Hill K.E. (2009). Selenoprotein P—Expression, functions, and roles in mammals. Biochim. Biophys. Acta.

[6] Olson G.E., Winfrey V.P., NagDas S.K., Hill K.E., Burk R.F. (2007). Apolipoprotein E receptor-2 (ApoER2) mediates selenium uptake from selenoprotein P by the mouse testis. J. Biol. Chem..

[7] Olson G.E., Winfrey V.P., Hill K.E., Burk R.F. (2008). Megalin mediates selenoprotein P uptake by kidney proximal tubule epithelial cells. J. Biol. Chem..

[8] Burk R.F., Hill K.E., Motley A.K., Austin L.M., Norsworthy B.K. (2006). Deletion of selenoprotein P upregulates urinary selenium excretion and depresses whole-body selenium content. Biochim. Biophys. Acta.

[9] Hill K.E., Zhou J., McMahan W.J., Motley A.K., Austin L.M., Gesteland R.F., Burk R.F. (2003). Deletion of selenoprotein P alters distribution of selenium in the mouse. J. Biol. Chem..

[10] Hill K.E., Zhou J., Austin L.M., Motley A.K., Ham A.J., Olson G.E., Atkins J.F., Gesteland R.F., Burk R.F. (2007). The selenium-rich *C*-terminal domain of mouse selenoprotein P is necessary for the supply of selenium to brain and testis but not for the maintenance of whole body selenium. J. Biol. Chem..

[11] Kurokawa S.E.S., Rose K.L., Wu S., Motley A.K., Hill S., Winfrey V.P., McDonald W.H., Capecchi M.R., Atkins J.F., Arnér E.S. (2014). Sepp1^UF^ forms are N-terminal selenoprotein P truncations that have peroxidase activity when coupled with thioredoxin reductase-1. Free Radic. Biol. Med..

[12] Du X.B., Li H.P., Wang Z., Qiu S., Liu Q., Ni J.Z. (2013). Selenoprotein P and selenoprotein M block Zn^2+^-mediated aβ_42_ aggregation and toxicity. Metallomics.

[13] Du X.B., Wang Z., Zheng Y.B., Li H.P., Ni J.Z., Liu Q. (2014). Inhibitory effect of selenoprotein p on Cu^+^/Cu^2+^-induced aβ_42_ aggregation and toxicity. Inorg. Chem..

[14] Lu T., Pan Y., Kao S.Y., Li C., Kohane I., Chan J., Yankner B.A. (2004). Gene regulation and DNA damage in the ageing human brain. Nature.

[15] Bellinger F.P., He Q.P., Bellinger M.T., Lin Y., Raman A.V., White L.R., Berry M.J. (2008). Association of selenoprotein P with Alzheimer’s pathology in human cortex. J. Alzheimers Dis..

[16] Takemoto A.S., Berry M.J., Bellinger F.P. (2010). Role of selenoprotein P in Alzheimer’s disease. Ethn. Dis..

[17] Lefevre J., Lefèvre J., Chernov K.G., Joshi V., Delga S., Toma F., Pastré D., Curmi P.A., Savarin P. (2011). The C terminus of tubulin, a versatile partner for cationic molecules: Binding of Tau, polyamines, and calcium. J. Biol. Chem..

[18] Hill K.E., Wu S., Motley A.K., Stevenson T.D., Winfrey V.P., Capecchi M.R., Atkins J.F., Burk R.F. (2012). Production of selenoprotein P (Sepp1) by hepatocytes is central to selenium homeostasis. J. Biol. Chem..

[19] Scharpf M., Schweizer U., Arzberger T., Roggendorf W., Schomburg L., Köhrle J. (2007). Neuronal and ependymal expression of selenoprotein P in the human brain. J. Neural. Transm..

[20] Steinbrenner H., Alili L., Bilgic E., Sies H., Brenneisen P. (2006). Involvement of selenoprotein P in protection of human astrocytes from oxidative damage. Free Radic. Biol. Med..

[21] Burk R.F., Hill K.E., Motley A.K., Winfrey V.P., Kurokawa S., Mitchell S.L., Zhang W. (2014). Selenoprotein P and apolipoprotein E receptor-2 interact at the blood-brain barrier and also within the brain to maintain an essential selenium pool that protects against neurodegeneration. FASEB J..

[22] Perez E.A. (2009). Microtubule inhibitors: Differentiating tubulin-inhibiting agents based on mechanisms of action, clinical activity, and resistance. Mol. Cancer Ther..

[23] Tanemura K., Murayama M., Akagi T., Hashikawa T., Tominaga T., Ichikawa M., Yamaguchi H., Takashima A. (2002). Neurodegeneration with Tau accumulation in a transgenic mouse expressing V337M human Tau. J. Neurosci..

[24] Ishihara T., Higuchi M., Zhang B., Yoshiyama Y., Hong M., Trojanowski J.Q., Lee V.M. (2001). Attenuated neurodegenerative disease phenotype in Tau transgenic mouse lacking neurofilaments. J. Neurosci..

[25] Zhang B., Maiti A., Shively S., Lakhani F., McDonald-Jones G., Bruce J., Lee E.B., Xie S.X., Joyce S., Li C. (2005). Microtubule-binding drugs offset Tau sequestration by stabilizing microtubules and reversing fast axonal transport deficits in a tauopathy model. Proc. Natl. Acad. Sci. USA.

[26] Brunden K.R., Zhang B., Carroll J., Yao Y., Potuzak J.S., Hogan A.M., Iba M., James M.J., Xie S.X., Ballatore C. (2010). Epothilone D improves microtubule density, axonal integrity, and cognition in a transgenic mouse model of tauopathy. J. Neurosci..

[27] Van Eersel J., Ke Y.D., Liu X., Delerue F., Kril J.J., Götz J., Ittner L.M. (2010). Sodium selenate mitigates Tau pathology, neurodegeneration, and functional deficits in Alzheimer’s disease models. Proc. Natl. Acad. Sci. USA.

[28] Song G.L., Zhang Z.H., Wen L., Chen C., Shi Q.X., Zhang Y., Ni J.Z., Liu Q. (2014). Selenomethionine ameliorates cognitive decline, reduces Tau hyperphosphorylation, and reverses synaptic deficit in the triple transgenic mouse model of Alzheimer’s disease. J. Alzheimers Dis..

[29] Zhao Y., Zhao B. (2013). Oxidative stress and the pathogenesis of Alzheimer’s disease. Oxid. Med. Cell Longev..

[30] Gamblin T.C., King M.E., Kuret J., Berry R.W., Binder L.I. (2000). Oxidative regulation of fatty acid-induced Tau polymerization. Biochemistry.

[31] Eckers J.C., Kalen A.L., Xiao W.S., Sarsour E.H., Goswami P.C. (2013). Selenoprotein P inhibits radiation-induced late reactive oxygen species accumulation and normal cell injury. Int. J. Radiat. Oncol. Biol. Phys..

[32] Xiao W.S., Zhu Y., Sarsour E.H., Kalen A.L., Aykin-Burns N., Spitz D.R., Goswami P.C. (2013). Selenoprotein P regulates 1-(4-Chlorophenyl)-benzo-2,5-quinone-induced oxidative stress and toxicity in human keratinocytes. Free Radic. Biol. Med..

[33] Tian J., Liu Q., Dong S., Qiao X.F., Ni J.Z. (2010). A new method for multi-site-directed mutagenesis. Anal. Biochem..

